# Safe Anesthetic Management of One-Lung Ventilation in an Adolescent Patient With Fontan Circulation: A Case Report

**DOI:** 10.7759/cureus.70353

**Published:** 2024-09-27

**Authors:** Masaaki Kawakami, Hisakatsu Ito, Tomonori Takazawa

**Affiliations:** 1 Department of Anesthesiology, Faculty of Medicine, University of Toyama, Toyama, JPN

**Keywords:** congenital heart disease, fontan circulation, general anesthesia, one-lung ventilation, pulmonary vascular resistance

## Abstract

We present a case report on anesthesia management for a 16-year-old male with Fontan circulation undergoing pacemaker implantation, necessitating one-lung ventilation. The patient had a complex congenital heart condition and had previously undergone multiple surgeries, including a Fontan procedure. Due to his unique cardiovascular anatomy, a left thoracotomy was chosen for pacemaker implantation, which required one-lung ventilation for surgical approach and visualization. This procedure can pose significant risks in patients with Fontan circulation due to potential intraoperative increases in pulmonary vascular resistance, which is notoriously difficult to manage in the acute setting. Preoperative assessments showed that the patient had good exercise tolerance, preserved biventricular contractility, and normal liver and kidney function. However, he had a slightly elevated central venous pressure. We employed a meticulous anesthetic plan to minimize the risks associated with one-lung ventilation. This included inserting pre-induction arterial and central venous catheters, using total intravenous anesthesia with propofol and remifentanil, and administering circulatory support agents, such as dobutamine and norepinephrine. Arterial blood and central venous pressure were closely monitored throughout the procedure. Further, cardiac activity was monitored using transesophageal echocardiography (TEE). The patient's hemodynamic stability was maintained intraoperatively, and the pacemaker lead was successfully implanted without significant complications. Postoperatively, we administered continuous intravenous fentanyl infusion and intercostal nerve blocks for pain control. He had an uneventful recovery and was discharged from the intensive care unit the day after surgery. This case contributes to the limited but growing body of literature on anesthesia management in patients with Fontan circulation, particularly related to the use of one-lung ventilation, and appropriate selection of anesthetic agents and circulatory support drugs. Further research is necessary to establish best practices and improve surgical outcomes in these patients.

## Introduction

Advances in the treatment of congenital heart disease have led to an increasing number of long-term survivors [1]. In particular, more patients survive to adulthood with Fontan circulation [2]. Consequently, there are more reports of general anesthesia in patients with Fontan circulation, including surgical cases with highly variable hemodynamics, such as patients presenting for cesarean section and pheochromocytomas [3-5]. In patients with Fontan circulation, venous blood returning from the body circulation flows directly to the pulmonary artery; therefore, circulatory dynamics in these patients are sensitive to respiratory status [1]. Performing one-lung ventilation in patients with Fontan circulation poses the risk of circulatory collapse due to increased pulmonary vascular resistance as a result of both the Fontan circulation and one-lung ventilation. Multiple factors, including hypoxic pulmonary vasoconstriction, hypercarbia, and increased airway pressure, can cause increased pulmonary vascular resistance. While a successful case of one-lung ventilation in a patient with Fontan circulation was recently reported, general anesthesia management for such a case remains challenging [6-8]. In this case report, we share our experience of safely conducting pacemaker implantation under general anesthesia with one-lung ventilation in the lateral position for a patient with Fontan circulation.

## Case presentation

The patient was a 16-year-old male with dextrocardia who was born with a double inlet right ventricle, double outlet right ventricle, and modified great artery transposition. His height and weight were 169 cm and 69 kg, with a body mass index of 24. He had undergone bidirectional Glenn surgery and the Damus-Kaye-Stansel procedure at one year old, followed by a Fontan procedure with extracardiac total cavopulmonary connection at two years old. The patient developed sick sinus syndrome at 12 years old but has had no circulatory compromise since then. However, he had to relocate to a distant location, and a pacemaker insertion was scheduled.

**Figure 1:**
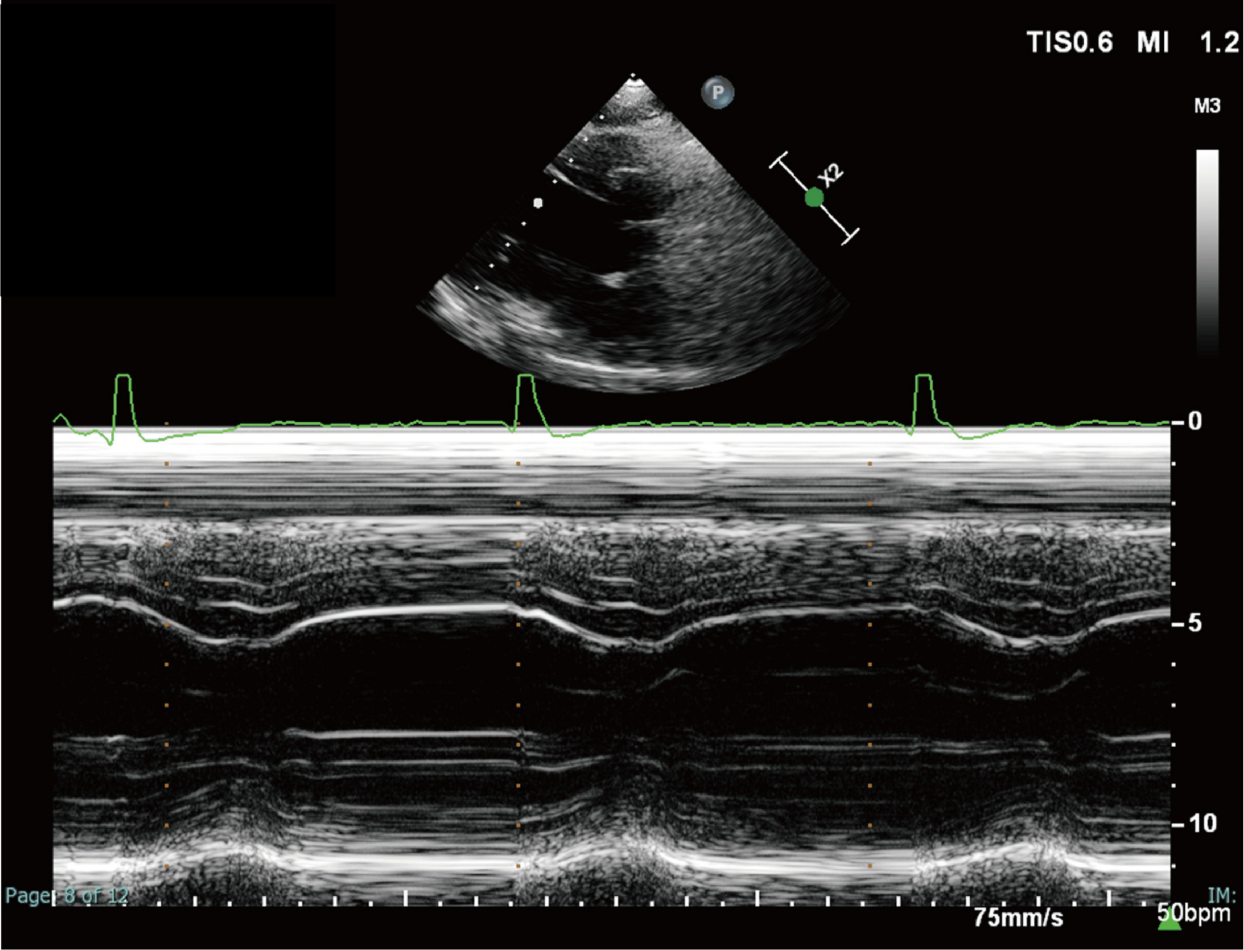
Preoperative echocardiogram M-mode echocardiography shows a long-axis cross-sectional image of the ventricle at the level of the tendon cords. The ultrasound probe was placed on the left margin of the fourth sternal rib. Left ventricular end-diastolic and -systolic diameter was 50 and 33 mm, respectively. Left ventricular ejection fraction and right ventricular area change were 63% and 35%, respectively. All of these measurements were within normal limits.

The patient had sufficient exercise tolerance to be able to play basketball. Echocardiography revealed preserved biventricular contractility (Figure 1) (Video 1).

**Video 1:** Preoperative echocardiogram The movie shows a short-axis image of the ventricle at the level of the tendon cords. A good ventricular contraction is confirmed.

Blood tests indicated a hemoglobin level of 18.3 g/dL, with liver and kidney function within normal ranges. Preoperative catheterization showed satisfactory blood flow through the extracardiac conduit, with cardiac output and cardiac index at 4.44 and 2.90 L/min, respectively, both within their normal ranges. The pulmonary artery resistance/systemic artery resistance ratio was within the normal range at 0.04, while the central venous pressure was mildly elevated at 14 mmHg (Table 1).

**Table 1:** Preoperative cardiac catheterization data CO: cardiac output; CI: cardiac index; Rp/Rs: pulmonary artery resistance/systemic artery resistance ratio; Qp/Qs: pulmonary blood flow/systemic blood flow ratio; VO2: oxygen uptake; HR: heart rate; E/A: early diastolic flow velocity/late diastolic flow velocity ratio; RVFAC: right ventricular fractional area change; CVP: central venous pressure.

Parameters	Observed Values
CO (L/min)	4.44
CI (L/min/m^2^)	2.90
Rp/Rs	0.04 (0.68/16.89)
Qp/Qs	1.00 (4.44/4.44)
VO_2_ (ml/min/m^2^)	131
HR (bpm)	61
E/A	2.04 (0.49/0.24)
RVFAC (%)	40
CVP (cmH_2_O)	14

Due to the difficulty in transvenous access owing to his cardiovascular anatomy, a left thoracotomy approach was chosen to implant the pacemaker lead directly onto the atrial wall. Consequently, one-lung ventilation was needed during the intraoperative management. However, we were concerned about the disruption of the Fontan circulation due to increased pulmonary vascular resistance, particularly from the one-lung ventilation. To address this issue, we inserted arterial and central venous catheters under awake conditions in advance and carefully induced general anesthesia while administering circulatory support agents under arterial and central venous pressure monitoring. We prepared for the nitric oxide inhalation and the immediate introduction of extracorporeal membrane oxygenation (ECMO) to deal with the eventuality of circulatory collapse during anesthesia. For example, if the central venous pressure increased significantly with positive pressure ventilation or one-lung ventilation, we planned to introduce ECMO. Further, percutaneous pacing was on standby in the room where the surgery was performed.

**Figure 2:**
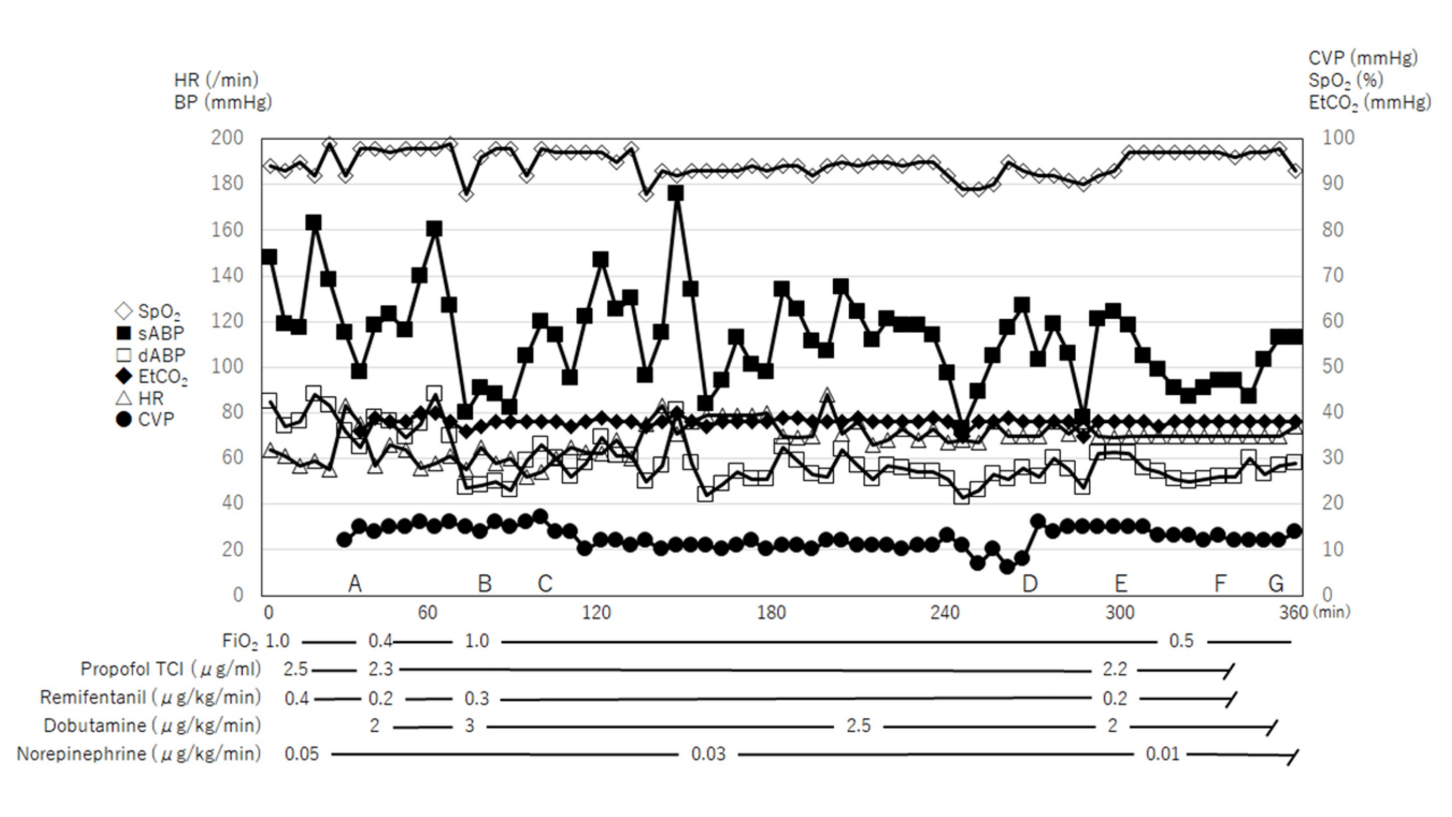
Intraoperative hemodynamic and respiratory changes during the surgery No major hemodynamic deterioration was observed throughout the operation. A: intubation; B: start of one-lung ventilation (OLV); C: start of operation; D: start of cardiac pacing at a frequency of 80 beats/minute in AAI mode; E: end of OLV; F: end of operation; G: extubation. HR: heart rate; BP: blood pressure; CVP: central venous pressure; SpO_2_: percutaneous oxygen saturation; EtCO_2_: partial pressure of carbon dioxide; sABP: systolic arterial blood pressure; dABP: diastolic arterial blood pressure; FiO_2_: fraction of inspired oxygen; TCI: target-controlled infusion.

General anesthesia was induced and maintained using total intravenous anesthesia with propofol and remifentanil. Dobutamine and norepinephrine were started before anesthesia induction and administered throughout the surgery (Figure 2).

The amount of vasopressor was adjusted under transesophageal echocardiography (TEE) observation of the movement of the left ventricle and the common atrioventricular valve, maintaining his systolic blood pressure between 100 and 140 mmHg. Consequently, dobutamine was administered at the rate of 2-3 μg/kg/minute and norepinephrine at 0.01-0.05 μg/kg/minute. The patient's trachea was intubated with a double-lumen endotracheal tube (32 Fr, Smiths Medical Japan, Japan), and pressure-controlled ventilation was performed. The controlled pressure was adjusted to maintain the end-tidal partial pressure of carbon dioxide (ETCO_2_) below 40 mmHg. We confirmed stable arterial blood pressure, central venous pressure, and TEE findings at the start of one-lung ventilation in the supine position. Once the patient was intraoperatively placed in the right lateral position, we needed to increase the controlled pressure to 20 cmH_2_O, and, based on the value of ETCO_2_, it needed to be increased to a maximum level of 30 cmH_2_O (Table 2).

**Table 2:** Changes in hemodynamic and respiratory parameters during the pre- and intraoperative period Since pressure-controlled ventilation was performed in this case, the airway pressure indicates the set value, while the tidal volume indicates the measured value. Positive end-expiratory pressure was set at 5 cmH_2_O during mechanical ventilation. HR: heart rate; BP: blood pressure; CVP: central venous pressure; ND: no data; SpO_2_: percutaneous oxygen saturation.

Parameters	Pre-anesthesia Induction	Post-anesthesia Induction	One-Lung Ventilation in Supine Position	One-Lung Ventilation in Lateral Position
HR (beats/min)	65	64	61	60
BP (mmHg)	148/65	116/69	127/70	130/60
CVP (mmHg)	13	14	16	16
Airway pressure (cmH_2_O)	ND	16	15	20
Respiratory rate (breaths/minute)	ND	12	15	15
Tidal volume (mL)	ND	400	350	350
End-tidal CO_2_ (mmHg)	ND	39	38	39
SpO_2_ (%)	96	98	99	98

Although the arterial partial pressure of carbon dioxide (PaCO_2_) was in the 50 mmHg range, and the lowest arterial partial pressure of oxygen (PaO_2_) was approximately 70 mmHg during one-lung ventilation (Table 3), there were no significant deteriorations in hemodynamics and respiratory status throughout the surgery. The pacemaker lead was successfully implanted into the left atrium as planned (Figure 1).

**Table 3:** Perioperative blood gas analysis results PaCO_2_: partial pressure of carbon dioxide; EtCO_2_: partial pressure of end-tidal carbon dioxide; ND: no data; PaO_2_: partial pressure of arterial oxygen; FiO_2_: fraction of inspired oxygen; P/F ratio: PaO_2_/FiO_2_ ratio; A-aDO_2_: alveolar-arterial oxygen difference; HCO_3_^-^: bicarbonate ion; BE: base excess.

Parameters	Preoperative Cardiac Catheterization	One-Lung Ventilation	Pacemaker Implantation	ICU Admission
pH	7.38	7.26	7.29	7.35
PaCO_2 _(mmHg)	40.4	55.0	51.9	44.4
EtCO_2 _(mmHg)	ND	36.0	35.0	ND
PaO_2 _(mmHg)	77.0	86.0	69.5	74.5
FiO_2_	0.21	1.0	1.0	0.5
P/F ratio	366	86	70	149
A-aDO_2 _(mmHg)	ND	558	578	156
HCO_3_^-^ (mmol/L)	23.2	23.9	24.1	23.6
BE (mmol/L)	ND	-4.0	-3.1	-1.9

The patient was transferred to the intensive care unit (ICU) after the removal of the endotracheal tube in the operating room. Continuous intravenous fentanyl infusion at a rate of 50 μg/hour and intercostal nerve block with 0.25% levobupivacaine at 5 mL/hour were administered for postoperative pain management. Since the patient's postoperative course was uneventful, he was discharged from the ICU on the first postoperative day. Eight days after surgery, he walked out of the hospital on his own.

## Discussion

Here, we describe the successful management of one-lung ventilation for pacemaker implantation via a lateral thoracotomy incision in an adolescent male with Fontan circulation. Despite the anticipated risk of circulatory collapse due to restricted pulmonary blood flow during one-lung ventilation, we successfully performed safe general anesthesia management using vasopressors under careful circulatory and respiratory monitoring.

Bradycardic arrhythmias associated with atrial dysfunction occur frequently as a remote complication of Fontan surgery, increasing the likelihood of the need for pacemaker implantation [9]. Due to the difficulty of transvenous access owing to Fontan anatomy and considering the chest adhesions caused by repeated cardiac surgeries, a lateral thoracotomy approach was the preferred surgical technique in our patient [10]. In such cases, thorough preoperative evaluation regarding the feasibility of one-lung ventilation is crucial. One-lung ventilation poses the risk of circulatory collapse for patients with Fontan circulation due to the potential for an increase in pulmonary vascular resistance secondary to hypoxemia, hypercapnia-induced pulmonary vasoconstriction, and elevated intrathoracic pressure [8,11]. In this case, we tried to ensure that the minute ventilation was as high as possible without the airway pressure exceeding 30 cmH_2_O while preventing the PaCO_2_ from becoming too high. In general, any attempt to lower PaCO_2_ decreases preload due to an increase in intrathoracic pressure. Although not limited to this case, one-lung ventilation in patients with Fontan circulation requires careful ventilator setting [11].

Cases of circulatory collapse resulting from high pulmonary vascular resistance in patients with Fontan circulation, which leads to an increase in central venous pressure and a decrease in blood pressure, have been previously reported [12,13]. On the other hand, there are reports of cases in which one-lung ventilation during surgery did not result in circulatory collapse [6-8,14]. However, since the perioperative requirements to avoid circulatory collapse during noncardiac surgery in patients with Fontan circulation have not been established, more such cases need to be accumulated. In the current case, the patient tolerated high-impact aerobic exercise, such as basketball, before pacemaker implantation. Additionally, his ventricular ejection function was preserved, and both central venous pressure and pulmonary vascular resistance were low and well-maintained. These facts served as predictive factors for his likely tolerance to the increase in pulmonary vascular resistance associated with positive pressure ventilation and one-lung ventilation. This suggests that patients with Fontan circulation with maintenance of ventricular contractility and with a lower established central venous pressure, as seen in this case, might have a higher ability to withstand positive pressure respiratory management under one-lung ventilation. Even in such cases, the central venous and airway pressure should be strictly controlled to stabilize the circulation, as we did in this case.

We selected total intravenous anesthesia with propofol for anesthesia in this case. Previous reports suggested that propofol does not suppress pulmonary vasoconstriction induced by hypoxemia and hypercapnia, in contrast to inhalation anesthetics [15]. The little available evidence from randomized controlled trials suggests differences in outcomes with anesthesia maintained using intravenous versus inhalational anesthesia during one-lung ventilation in normal circulation cases [16]. However, considering the potentially lethal impact of increased pulmonary vascular resistance on circulation in Fontan circulation, it is essential to investigate the extent to which the choice of anesthetic agents might influence outcomes. Future research is needed to address this uncertainty.

In this case, we administered dobutamine and norepinephrine as circulatory support agents. Low doses of norepinephrine and vasopressin are considered helpful in maintaining arterial blood pressure without increasing pulmonary vascular resistance [17]. Additionally, the increase in cardiac output with dobutamine is believed to be beneficial in countering circulatory suppression induced by general anesthesia and positive pressure ventilation.

Phosphodiesterase type III inhibitors are reportedly effective in reducing pulmonary vascular resistance. However, it remains unclear which circulatory support drugs contribute to postoperative outcomes in patients with Fontan circulation. We did not use phosphodiesterase type III inhibitors in this case because they reduce both pulmonary and systemic vascular resistance. Instead, we prepared nitric oxide on standby in case oxygenation could not be maintained or circulatory failure due to positive pressure ventilation was suspected [11,18].

Postoperatively, we were able to extubate the patient in the operating room, thus minimizing the time spent on positive pressure ventilation, which is detrimental for patients with Fontan circulation. We also paid attention to postoperative pain control to normalize breathing patterns and avoid significant respiratory depression [11].

## Conclusions

We could safely conduct general anesthesia with one-lung ventilation in the lateral position for a patient with Fontan circulation. One-lung ventilation might be feasible even in Fontan circulation patients when exercise tolerance, cardiac function, and low pulmonary vascular resistance are preserved. Adequate circulatory support, including catecholamine infusion, and continuous monitoring of cardiac function by TEE are required for safe anesthetic management in such cases. Preparing ECMO and percutaneous pacing is also essential in cases of circulatory collapse.
